# Nasal Airflow Measured by Rhinomanometry Correlates with FeNO in Children with Asthma

**DOI:** 10.1371/journal.pone.0165440

**Published:** 2016-10-28

**Authors:** I-Chen Chen, Yu-Tsai Lin, Jong-Hau Hsu, Yi-Ching Liu, Jiunn-Ren Wu, Zen-Kong Dai

**Affiliations:** 1 Department of Pediatrics, Kaohsiung Medical University Hospital, Kaohsiung, Taiwan; 2 Department of Otolaryngology, Kaohsiung Chang Gung Memorial Hospital and Chang Gung University College of Medicine, Kaohsiung, Taiwan; 3 Department of Pediatrics, School of Medicine, College of Medicine, Kaohsiung Medical University, Kaohsiung, Taiwan; National and Kapodistrian University of Athens, GREECE

## Abstract

**Background:**

Rhinitis and asthma share similar immunopathological features. Rhinomanometry is an important test used to assess nasal function and spirometry is an important tool used in asthmatic children. The degree to which the readouts of these tests are correlated has yet to be established. We sought to clarify the relationship between rhinomanometry measurements, fractional exhaled nitric oxide (FeNO), and spirometric measurements in asthmatic children.

**Methods:**

Patients’ inclusion criteria: age between 5 and 18 years, history of asthma with nasal symptoms, and no anatomical deformities. All participants underwent rhinomanometric evaluations and pulmonary function and FeNO tests.

**Results:**

Total 84 children were enrolled. By rhinomanometry, the degree of nasal obstruction was characterized as follows: (1) no obstruction in 33 children, (2) slight obstruction in 29 children, and (3) moderate obstruction in 22 children. FeNO was significantly lower in patients without obstruction than those with slight or moderate obstruction. Dividing patients according to ATS Clinical Practice Guidelines regarding FeNO, patients < 12 years with FeNO > 20 ppb had a lower total nasal airflow rate than those with FeNO < 20 ppb. Patients ≥ 12 years with FeNO > 25 ppb had a lower total nasal airflow rate than those with FeNO < 25 ppb.

**Conclusions:**

Higher FeNO was associated with a lower nasal airflow and higher nasal resistance. This supports a relationship between upper and lower airway inflammation, as assessed by rhinomanometry and FeNO. The results suggest that rhinomanometry may be integrated as part of the functional assessment of asthma.

## Introduction

Epidemiological and clinical studies suggest a relationship between rhinitis and asthma, because both conditions have a similar pathogenesis and basic immunology [1, 2]. One study found the comorbidity of rhinitis and asthma to be as high as 100% [2]. Local allergen exposure in the nose of patients with allergic rhinitis can quickly lead to asthma or significant allergic inflammation in the lung [3], with an elevation of fractional exhaled nitric oxide (FeNO) [4]. It is important for pediatricians to understand the relationship between these two disorders that all children with rhinitis are evaluated for lower airway disease, and all patients with asthma are screened for upper airway conditions.

Rhinomanometry is a simple and useful test that objectively evaluates nasal airway patency [5, 6], involving simultaneous measurements of nasal airway resistance, nasal airflow, and transnasal pressure [7, 8]. In the clinical setting, it is often used to diagnose nasal obstruction and to follow patients treated with medical and surgical procedures to improve nasal patency [9, 10]. Moreover, it can be useful for observing seasonal allergic rhinitis [11], studying nasal hyperresponsiveness [12–14], evaluating various intranasal treatments [15, 16], and for the follow-up of nasal provocation testing with allergic response mediators [17, 18]. The measurement of unilateral nasal resistance also detects anatomical obstructions and helps evaluate the efficacy of corrective surgery.

FeNO is a small molecule produced by human secretory cells, and is now generally recognized as a marker of airway inflammation [19]. FeNO may increase during asthma and airway hyperresponsiveness, and decrease with anti-inflammatory treatments, such as corticosteroids [20]. Furthermore, symptoms of rhinitis may be partly responsible for increased FeNO, independently of asthma control [4]. FeNO is now generally considered a valuable biomarker in allergic airway disease.

The most likely connection between allergic rhinitis and asthma is that they have the same underlying immunopathology, probably through a type I IgE hypersensitivity reaction with a unified systemic immunological reaction to allergens. The pathophysiological relationship between the upper and lower airways is now well established in adults [21]. However, currently the extent of relationship between asthma control and allergic rhinitis in children is unclear. Moreover, the present correlation between rhinitis and asthma is mostly based on epidemiologic findings [1,2], and an objective and functional evaluation to link upper and lower airway is not yet established. To our knowledge, only one previous report mentioned an association between upper and lower airway patency by acoustic rhinomanometry and spirometry [22], and relationship among FeNO and measurements of rhinomanometry and spirometry has never been reported.

Here we investigated the relationship between rhinomanometry measurements, including nasal resistance and nasal airflow, FeNO, spirometric measurement, and IgE levels in asthmatic children. Besides, plasma IgE and allergic rhinitis symptom scores were also evaluated.

## Materials and Methods

### Subjects

Subjects were selected from visitors to the pediatric cardiopulmonary clinic of Kaohsiung Medical University from January 2014 to August 2015 for evaluation of asthma. Patients who met the following criteria were eligible for inclusion: age between 5 and 18 years, and previously or newly diagnosed with asthma, without receiving any inhaled or systemic corticosteroid, nasal or oral antihistamine, or decongestant in recent one week. Asthma was defined according to the Global Initiative for Asthma (GINA) [23]. Those who had a fever or acute rhinosinusitis within the previous two weeks and those with anatomical deformities causing airway obstruction, such as a tumor, a polyp, or choanal atresia, were excluded. A detailed medical history was obtained for all participants, and information regarding their age, gender, allergic rhinitis symptoms, disease duration, family history of atopy, and comorbid conditions were recorded.

The protocol for this study was approved by the institutional review board of Kaohsiung Medical University (KMUH-IRB20140104). Written informed consent was obtained from the parents of all children.

### Rhinomanometric evaluations

Nasal flow and resistance in all patients were evaluated using anterior rhinomanometry (Jaeger Master Scope Rhino; Jaeger GmbH, Würzburg, Germany). Each patient was allowed to rest for 20 min at room temperature (25°C) before being requested to maintain an upright sitting position. After placing a nasal probe into either nostril, the participant was asked to breathe through one nostril with a closed mouth, during which transnasal flow and pressure measurements were recorded using a computer. The average of three consecutive nasal cavity flow measurements was recorded as the final result. All measurements were made using a steady pressure of 150 Pa, as recommended by the European Rhinomanometry Standardization Committee [24]. Ranges of nasal flow have been correlated with different levels of breathing comfort in the general population. Reference ranges of rhinomanometry for airway values for various degrees of nasal obstruction symptoms are known (at a pressure of 150 Pa) [25, 26]. The degree of nasal obstruction was estimated in terms of total nasal flow, corresponding to no obstruction (> 800 cm^3^/s), slight obstruction (500-800 cm^3^/s), moderate obstruction (300–500 cm^3^/sec), severe obstruction (100–300 cm^3^/s), and airway closed (< 100 cm^3^/s) [26].

### FeNO measurements

FeNO was measured using a Nano Coulomb nitric oxide analyzer (Shangwo Biotechnology Co., Ltd., Jiangsu, China) following its instructions and American Thoracic Society (ATS) recommendations; results are expressed in parts per billion (ppb) [27, 28]. Briefly, patients were tested in a resting state. After inhalation of ambient air through a nitric oxide scrubber to total lung capacity, patients then exhaled against expiratory resistance to exclude nasal air. The duration of exhalation must be sufficient (at least 6 s) to obtain a plateau in the NO versus time profile of at least 3 s in children > 12 years old and a 2s plateau duration in children less than 12 years old. Repeated exhalations (two values that agreed within 5% or three that agreed within 10%) were performed at a constant flow rate of 50 mL/s. FeNO was measured on the same day as the spirometry and rhinomanometry.

### IgE measurements

Blood samples were collected in a heparin-containing venipuncture tube. Total plasma IgE was measured using a microparticle enzyme immunoassay (IMMULITE 2000 XPi Immunoassay System, Siemens Healthineers, USA).

### Pulmonary function test

Spirometric evaluations were performed in all participants using a body plethysmograph (Jaeger Masterscreen Body, Würzburg, Germany) at room temperature with patients in an upright sitting position using a nose clip. The best values of at least three consecutive measurements were recorded for each participant. Recorded measurements included forced vital capacity (FVC), forced expiratory volume in 1 s (FEV_1_), mid-flow rate/forced expiratory flow at 25–75% of FVC (FEF25-75), peak expiratory flow (PEF), and FEV_1_/FVC. Results are expressed as percentages of predicted values.

### Allergic rhinitis symptom scores questionnaire

Information on current and past allergic symptoms and diagnoses of allergic diseases was collected using a modified International Study and Asthma and Allergies Childhood (ISAAC) standard written questionnaire [29]. Written questionnaires were completed by the children’s parents and a total symptom score (TSS) was determined.

### Statistical analyses

Statistical analyses were performed using SPSS software (ver. 15; SPSS Inc., Chicago, IL, USA). Spirometric and rhinomanometric data are presented as means and standard deviations for all groups. Data were compared between groups using two-tailed unpaired *t*-tests. Correlations between variables were evaluated by Pearson’s coefficient. A p value < 0.05 was considered to indicate statistical significance.

## Results

### Patient characteristics

We enrolled 84 patients during the study period. Demographic, FeNO, rhinomanometric and spirometric measurements, and serum total IgE level are shown in Table 1.

**Table 1:** Characteristics of the study population.

	Patients (*n* = 84)	
	Mean (± SD)	Range
Age (years)	9.2 (± 4.0)	5–18
Male gender (%)	58.3	
BMI (kg/m^2^)	18.3 (±4.0)	11.8–23.5
BSA (m^2^)	1.14 (± 0.33)	0.66–1.90
Total flow (cm^3^/s)	755.2 (± 337.8)	321.8–1704.8
Resistance (150 Pa/cm^3^/s)	0.25 (± 0.14)	0.09–0.47
FeNO (ppb)	21.9 (± 13.2)	2.2–57.7
FVC (%)	104.7 (± 65.2)	29.7–295.5
FEV_1_/FVC (%)	108.3 (± 69.9)	36.3–503
PEF (%)	97.6 (± 48.9)	9.9–336.2
IgE (IU/mL)	562.4 (± 827.6)	2.1–3485

BMI: body mass index, BSA: body surface area, FeNO: fractional exhaled nitric oxide, FVC: forced vital capacity, FEV_1_: forced expiratory volume in 1 s, PEF: peak expiratory flow. *p* values < 0.05 were considered to indicate statistical significance.

#### Spirometric measurements and FeNO in patients with differing nasal obstruction severity

Patients were divided into three groups: no obstruction, with nasal total flow > 800 cm^3^/s in 33 (39.3%) children, slight obstruction with nasal flow of 500-800 cm^3^/s in 29 (34.5%) children, and moderate obstruction with nasal flow 300-500 cm^3^/s in 22 (26.2%) children. No patients had severe nasal obstruction or an airway closed. There were no significant differences between the groups in terms of age (p = 0.533), gender distribution (p = 0.382), body mass index (BMI) (p = 0.532), or BSA (p = 0.853; Table 2). FeNO tests demonstrated that FeNO levels in patients with slight or moderate nasal obstruction were significantly higher than in those with no nasal obstruction (p < 0.001; Table 2). There were no significant differences among the three groups in terms of FVC, FEV_1_, or PEF. Because of the significant differences in FeNO and total nasal flow as measured by rhinomanometry, patients were further divided into two groups according to the official ATS clinical practice guidelines for FeNO: age < 12 years (73.8%) and age ≥ 12 years (26.2%). BMI and BSA in patients aged < 12 years with moderate nasal obstruction were significantly lower than in those with no nasal obstruction or slight nasal obstruction (p = 0.006 and 0.004, respectively), and FeNO in patients with slight or moderate nasal obstruction was significantly higher than in those with no nasal obstruction (p < 0.001; Table 3). In patients aged ≥ 12 years, only FeNO in patients with slight or moderate nasal obstruction groups was significantly higher than in those with no nasal obstruction (p < 0.001; Table 3).

**Table 2:** Spirometric and rhinomanometric measurements in patients with differing severities of nasal obstruction.

Severity of nasal obstruction				
	No	Slight	Moderate	
Total nasal flow (cm^3^/s)	> 800	500–800	300–500	
*n*	33	29	22	
	Mean (± SD)	Mean (± SD)	Mean (± SD)	p-value
Age (years)	9.1(± 4.9)	8.9 (± 3.4)	9.8(± 5.5)	0.533
Male gender (%)	54.5	56.7	65.0	0.382
BMI (kg/m^2^)	18.9 (± 3.5)	18.2 (± 3.5)	17.7(± 4.9)	0.532
BSA (m^2^)	1.15 (± 0.29)	1.15 (± 0.31)	1.10 (± 0.42)	0.853
FeNO (ppb)	10.7 (± 4.7)	24.2 (± 11.3)	35.8 (± 8.7)	< 0.001
FVC (%)	91.2 (± 43.8)	100.0 (± 36.9)	102.0 (± 41)	0.073
FEV_1_/FVC (%)	91.0 (± 41.7)	114.0 (± 59.1)	127.6 (± 57.2)	0.159
PEF	106.1 (± 55.8)	100.4 (± 49.1)	79.9 (± 31.1)	0.158

BMI: body mass index, BSA: body surface area, FeNO: fractional exhaled nitric oxide, FVC: forced vital capacity, FEV_1_: forced expiratory volume in 1 s, PEF: peak expiratory flow. *p* values < 0.05 were considered to indicate statistical significance.

**Table 3:** Spirometric and rhinomanometric measurements in patients differentiated by symptoms of nasal obstruction.

Severity of nasal obstruction				
	No	Slight	Moderate	
Total nasal flow (cm^3^/s)	> 800	500–800	300–500	
	Mean (± SD)	Mean (± SD)	Mean (± SD)	p-value
**Age < 12 years**				
*n*	26	22	14	
BMI (kg/m^2^)	18.4 (± 3.4)	17.6 (± 3.8)	14.9 (± 1.3)	0.006
BSA (m^2^)	1.04 (± 0.20)	1.03 (± 0.23)	0.82 (± 0.15)	0.004
FeNO (ppb)	11.1 (± 4.7)	24.3 (± 11.7)	38.6 (± 9.0)	<0.001
FVC (%)	84.8 (± 41.7)	96.2 (± 23.8)	98.8 (± 44.1)	0.231
FEV_1_/FVC (%)	88.4 (± 38.1)	117.5 (± 88.3)	107.3 (± 44.9)	0.285
PEF	109.7 (± 56.8)	100.9 (±54.5)	84.6 (± 24.6)	0.382
**Age ≥ 12 years**				
*n*	7	7	8	
BMI (kg/m^2^)	20.6 (± 3.3)	19.8(± 2.2)	22.6 (±5.0)	0.365
BSA (m^2^)	1.57 (± 0.15)	1.53 (± 0.19)	1.60 (± 0.21)	0.765
FeNO (ppb)	9.3 (± 4.9)	24.0 (± 10.6)	30.9 (± 5.9)	<0.001
FVC (%)	113.9 (± 83.1)	111.8 (± 34.3)	113.3 (± 51.9)	0.338
FEV_1_/FVC (%)	100.2 (± 55.2)	103.1 (± 40.6)	158.1 (± 52.3)	0.347
PEF (%)	93.3 (± 54.2)	99.0 (± 28.9)	72.8 (± 39.8)	0.458

BMI: body mass index, BSA: body surface area, FeNO: fractional exhaled nitric oxide, FVC: forced vital capacity, FEV_1_: forced expiratory volume in 1 s, PEF: peak expiratory flow. *p* values < 0.05 were considered to indicate statistical significance.

#### Relationship between FeNO levels and spirometric and rhinomanometric measurements

Following the ATS recommendations for interpreting low FeNO measurements, we found that FeNO > 20 ppb was associated with a lower total nasal airflow rate (p < 0.001) and higher nasal resistance at a given pressure of 150 Pa (p < 0.001) than FeNO < 20 ppb in patients younger than 12 years old (Table 4). Moreover, patients aged ≥ 12 years with FeNO > 25 ppb showed a lower total nasal airflow rate (p < 0.001) and higher resistance at a given pressure of 150 Pa (p = 0.026) than those with FeNO < 25 ppb (Table 5).

**Table 4:** Spirometric and rhinomanometric measurements in patients aged < 12 years.

	FeNO (ppb)		
Age < 12 years	< 20	≥ 20	
*n*	34	28	
	Mean (± SD)	Mean (± SD)	p-value
Total nasal flow (cm^3^/s)	995.5 (± 279.1)	505.8 (±127.1)	< 0.001
Nasal resistance (150 Pa/cm^3^/s)	0.16 (± 0.01)	0.32 (±0.09)	< 0.001
FVC (%)	91.1 (±20.7)	110.0 (±28.1)	0.129
FEV_1_/FVC (%)	91.2 (±33.9)	114.0 (±39.1)	0.141
PEF (%)	104.5 (±51.0)	97.2 (±52.0)	0.589

FeNO: fractional exhaled nitric oxide, FVC: forced vital capacity, FEV_1_: forced expiratory volume in 1 s, PEF: peak expiratory flow. *p* values < 0.05 were considered to indicate statistical significance.

**Table 5:** Spirometric and rhinomanometric measurements in patients aged ≥ 12 years.

	FeNO (ppb)		
Age ≥ 12 years	< 25	≥ 25	
*n*	12	10	
	Mean (± SD)	Mean (± SD)	p-value
Total nasal flow (cm^3^/s)	919.6 (± 340.4)	438.7 (±133.2)	< 0.001
Nasal resistance (150 Pa/cm^3^/s)	0.19 (± 0.08)	0.41 (± 0.26)	0.026
FVC (%)	112.8 (± 46.1)	125.5 (± 48.1)	0.274
FEV_1_/FVC (%)	102.7 (± 50.5)	125.5 (± 56.3)	0.291
PEF (%)	95.6 (± 45.4)	78.0 (± 37.0)	0.328

FeNO: fractional exhaled nitric oxide, FVC: forced vital capacity, FEV_1_: forced expiratory volume in 1 s, PEF: peak expiratory flow. *p* values < 0.05 were considered to indicate statistical significance.

#### Association between allergic rhinitis symptoms questionnaire, IgE, and rhinomanometry

The allergic rhinitis symptoms questionnaire revealed significant differences in total nasal flow, plasma IgE level, and total symptom score (TSS) in subjects whose parents had a history of allergy (p = 0.037, 0.048, and < 0.001, respectively; Table 6).

**Table 6:** Family history of allergy from the allergic rhinitis questionnaire survey.

	Parents’ history of allergies		
	Positive	Negative	
*n*	42	33	
	Mean (± SD)	Mean (± SD)	p-value
Total nasal flow (cm^3^/s)	673.1 (± 283.6)	834.3 (±373.7)	0.037
Nasal resistance (150 Pa/cm^3^/s)	0.28 (± 0.17)	0.21 (± 0.09)	0.058
IgE (IU/mL)	759.2 (± 916.5)	331.5 (±651.5)	0.048
TSS	10.8 (± 2.6)	6.9 (± 2.9)	< 0.001

TSS: total symptom score. *p* values < 0.05 were considered to indicate statistical significance.

### Correlations

There was no significant correlation between total nasal flow and plasma IgE (r = -0.027; p = 0.543), but IgE was significantly correlated in a linear fashion with TSS (r = 0.226; p = 0.036). There was no significant correlation between TSS and total nasal flow (r = -0.069; p = 0.339) or nasal resistance (r = 0.022; p = 0.575).

## Discussion

Asthma and allergic rhinitis share numerous common pathophysiological features and primary immune responses to a local allergic stimulus, followed by a generalized airway reaction [30–32]. Previously, various methods, such as spirometry and FeNO measurements, have been used to assess the efficacy of asthma treatments. However, studies have shown that asthma is still not fully controlled in many patients [33, 34]. Despite environmental and allergic factors and poor adherence to treatment, rhinitis has been recognized as a cause of poor asthma control, and the development of rhinitis may be an indicator of persistent asthma [4, 35]. To date, there are few objective tools for evaluating rhinitis. FeNO may increase in rhinitis, independently of the control of asthma [4], and rhinomanometry allows objective assessment of nasal patency. Chawes et al. firstly found a strong and consistent association between upper and lower airway patency by acoustic rhinomanometry and spirometry in 221 six-year-old asthmatic children [22]. However, no other relationship between rhinomanometry measurements, FeNO, and spirometry measurements has been reported. For the first time, we studied correlations among rhinomanometry and spirometry measurements and FeNO and found that total nasal airflow and resistance measured by rhinomanometry correlate significantly with FeNO in asthmatic children. Allergic airway disease should be assessed by integrating these methods to improve the assessment and control of disease.

Regarding the relationship between FeNO and spirometry, Stănciulescu et al. performed a study in 89 children and found that FeNO did not correlate with FEV_1_ or FVC, but did correlate with MEF25 and PEF [36]. In the ATS recommendations for interpreting low FeNO measurements, the normal value is < 20 ppb in patients < 12 years of age and < 25 ppb in patients ≥ 12 years of age, indicating non-eosinophilic or no airway inflammation [27]. We found that FeNO levels were not significantly associated with spirometry results, consistent with a previous study [36]. Further studies and larger numbers of patients are needed to establish the relevance of FeNO to the evaluation of airflow obstruction by spirometry.

In adults, normal nasal resistance under congested nasal mucosal conditions is 0.25 Pa/cm^3^/s [37]. However, there are few reports on normal values of nasal resistance and nasal airflow in children. One study of nasal resistance by rhinomanometry in Japanese children revealed the mean nasal resistance was 0.43 ± 0.50 Pa/cm³/s in normal children [38]. Nasal resistance was significantly higher in those with nasal disease than in healthy children (0.56 ± 0.75 vs. 0.36 ± 0.21 Pa/cm³/s), and resistance tended to decrease with age [38]. The mean nasal resistance in our study of asthmatic children was 0.25 ± 0.14 Pa/cm³/s, which may provide a reference value for nasal resistance in Asian asthmatic children. Another study in 192 Caucasian children and adolescents found that nasal inspiratory flow and resistance at a transnasal pressure of 150 Pa are associated with age and height [7]. Other reports have shown that reference values for rhinomanometry are related to age, body surface area, and BMI [39, 40]. We believe that nasal inspiratory flow and nasal resistance relate to anatomical increases in the size and diameter of the nasal cavity. Body length, body weight, and age are confounding factors that may influence nasal airflow and resistance. In our study, an interesting finding in patients aged < 12 years was that BMI and BSA in those with moderate obstruction were significantly lower than in those with no or slight obstruction. However, this was not seen in patients aged ≥ 12 years. A possible explanation is the rapid growth rate of the nasal cavity in patients aged < 12 years, and body length and body weight would also significantly influence the total nasal flow in children between 5 and 12 years old.

There was no significant correlation between total nasal flow and total IgE in our patients. Total IgE levels are extremely variable and not always associated with asthma severity or airflow limitation [41]. In terms of rhinomanometry, the response to allergen provocation also does not necessarily correlate with serum specific IgE [42]. We did find that IgE levels were increased significantly in patients with a parent with allergies. Moreover, a positive parent history of allergies was associated with decreased total nasal flow and increased total symptom score on the questionnaire. These results should remind pediatricians of the importance of parents’ allergy histories, which can impact their children’s nasal disease, subjectively and objectively.

A major limitation of our study is the number of cases, which was simply too small to determine the full spectrum of relationships among these parameters. Larger numbers of cases in prospective, randomized studies are needed to determine relationships between rhinomanometric and spirometric measurements, IgE, allergic rhinitis symptom scores, and FeNO. However, the present study provides preliminary results regarding the relationship between the upper and lower airways. As abnormalities in nasal patency are often associated with respiratory symptoms in pediatric patients, information on the degree of nasal patency is thus helpful in selecting decongestive, anti-allergic, anti-infectious, anti-inflammatory, and other therapies, and may help in the management of asthmatic children.

In conclusion, our study revealed a significant correlation between nasal airflow as measured by rhinomanometry and FeNO in asthmatic children. In addition to spirometry and FeNO, we thus consider rhinomanometry to be valuable and recommend that it be integrated as part of the functional assessment of asthma.

## Abbreviations

FeNO: Fractional exhaled nitric oxide
GINA: Global Initiative for Asthma
ATS: American Thoracic Society
BMI: Body mass index
BSA: Body surface area
FVC: Forced vital capacity
FEV_1_: Forced expiratory volume in 1 s
FEF25-75: Mid-flow rate/forced expiratory flow at 25–75% of FVC
PEF: Peak expiratory flow
ISAAC: International Study and Asthma and Allergies Childhood
TSS: Total symptom score
